# Cats vs. Dogs: The Efficacy of Feliway Friends^TM^ and Adaptil^TM^ Products in Multispecies Homes

**DOI:** 10.3389/fvets.2020.00399

**Published:** 2020-07-10

**Authors:** Miriam Rebecca Prior, Daniel Simon Mills

**Affiliations:** Animal Behaviour Cognition and Welfare Group, School of Life Sciences, University of Lincoln, Lincoln, United Kingdom

**Keywords:** appeasing pheromone, diffuser, cat, dog, social behavior, stress

## Abstract

Seven percent of UK households are estimated to own both a cat and a dog, despite a popular view that the two do not live well together. This is the first study to evaluate the effects of pheromone products Feliway Friends^TM^ and Adaptil^TM^ on cat-dog interactions, in homes where owners perceived the potential for improvement in the relationship between their cat and dog. A blinded parallel randomized trial design over a 6-week period was used to evaluate the effect of each of the two products, with 17 participants in each group completing the trial. Owners reported weekly on the frequency of 10 specific undesirable interactions and seven specific desirable interactions. Total undesirable and desirable interaction scores both showed significant linear contrasts over time (undesirable score decreased; desirable score increased). Undesirable interaction scores were significantly lower (with a very large effect size) during treatment compared with baseline. There were no significant differences between the two pheromone products in relation to these outcome measures. Adaptil^TM^ and Feliway Friends^TM^ were both associated with a significant decrease in: dog chasing cat/cat runs away; cat hiding from dog; cat/dog staring at the other; and dog barking at cat. With Adaptil^TM^ a significant increase was also seen in: friendly greeting and times spent relaxed in the same room. From baseline (Week 2) to the end of the study (Week 6) there was a significant improvement in owners' perception of dog relaxation in those participants who received Adaptil^TM^ and of cat relaxation in those participants who received Feliway Friends^TM^. Similarity in the core chemical structure of the appeasing pheromones might explain the main effects, whilst different species-specific additions may explain the product-appropriate species-specific increases in relaxation scores. Specific behavioral improvements seen with Adaptil^TM^ may reflect a greater calming of dogs in this group, reducing their interest in seeking interaction with cats in the same home and the tension in the cat as a result. In conclusion, both products appear to improve the cat-dog relationship and it would be beneficial to further study their use in combination and against placebo. If selecting one product Adaptil^TM^ may be preferable, unless there is a particular need to increase the cat's relaxation.

## Introduction

Cats and dogs are the most popular pets, with an estimated 8.5 million pet dogs and 8 million pet cats in residence in the UK: 24% of UK households have at least one dog and 17% have at least one cat (1). These figures are not exceptional with global estimates of household dog ownership reported in the USA at 36.1%, Costa Rica at 53%, Sydney, Australia at 33.4%; and Teramo, Italy at 33%, with cat ownership in the USA at 31.6%; Costa Rica at 15%, Sydney, Australia at 22.5% and Teramo, Italy at 15% (2–5). Multi-species households are also increasingly common with recent estimates suggesting: 7% of UK households own both a cat and a dog; likewise, 7.8% of residents of Sydney, Australia, owned both species; and 7% of the residents of Teramo, Italy, owned both species (4–6).

Despite the prevalence of multi-species households across the globe, there has been little research to date on cats' and dogs' relationships with one another. Feuerstein and Terkel (7) highlight the suspected difficulty inherent in cat-dog communication; a stereotype that has permeated popular culture and is exemplified in the media by shows such as Nickelodeon's “CatDog,” Warner Bros. “Cats & Dogs” (2001), and Spike the dog in MGM's cartoon “Tom and Jerry”. Challenges in the cat-dog relationship can arise as a result of their differing behavioral tendencies and differences in typical social structure, for example cats lack a widely understood deference signal unlike dogs, and appear to place little importance on any observed hierarchy when determining access to key resources (8). Cats may also be seen as prey to some dogs, particularly those bred for hunting smaller mammals such as sighthounds or terriers. Feuerstein and Terkel (7) identified four behavioral categories in which the conveyed message is contradictory when performed by a cat compared with a dog: the horizontal full tail wag; stretching out forefeet; laying on back; and turning head away, all of which they consider to be appeasing/submissive signals in dogs but signs of frustration/aggression in cats. Despite these differences, cats and dogs are often able to form amicable relationships and most pet owners believe that their cat and dog are comfortable in each-other's presence (7, 9).

However, a poor relationship between a resident cat and dog can have serious consequences for the welfare of individual animals. There may be an unacceptable level of social stress or restricted access to key resources such as food, water or suitable toilet areas. There will also be increased stress for the remainder of the family (both human and animal), and potential risks of injury due to conflict. Importantly, it has been reported that a problematic relationship between a new pet and an existing pet is one of the top ten behavioral reasons for relinquishment to shelters for both cats and dogs (10). Whilst this latter study did not specify the percentage of cases in which problematic relationships were same-species or cat-dog relationships, the authors did specify that where cats were relinquished following acquisition of a dog in the previous year, they were much more likely to be relinquished for behavioral rather than non-behavioral reasons.

Pheromone-related products are believed to affect the emotional processing of animals that can detect them and are in widespread use as environmental adjuncts to aid in behavioral problems associated with stress and the perception of a stressful environment (11). In 1996, Feliway Classic^TM^, utilizing a synthetic version of the cat F3 facial-marking pheromone, was the first pheromone product for companion animals to be launched. Adaptil^TM^, based on the Dog Appeasing Pheromone (DAP) was launched in 2000, and Feliway Friends^TM^ (a.k.a Feliway MultiCat^TM^ in the USA) based on the Cat Appeasing Pheromone (CAP), was launched in 2016 (12).

The mammary region is the natural origin of the appeasing pheromone, being secreted from around 3–4 days after parturition until 2–5 days after weaning. Appeasing pheromones are believed to provide reassurance to offspring that persists even in the absence of a maternal figure (13). It is hypothesized that the presence of this mixture in the environment encourages offspring to remain calm while the mother hunts but also helps to identify safe areas to explore as the young develop and become more independent. The appeasing pheromones thus serve as an environmental signal that encourages a bias toward perceiving things as safe.

The different appeasing pheromone products (Adaptil^TM^ and Feliway Friends^TM^) contain some common fatty acids but also species-specific elements. Similar ratios of oleic acid, palmitic acid, and linoleic acid make up the generalized mammalian appeasing message, but it is suggested that additional species-specific components increase species-specific efficacy (13).

Evidence in support of the reassuring effect of DAP in dogs comes from data relating to a reduction in barking in shelter environments; separation related behaviors; firework fears; and anxiety at the veterinary clinic; as well as improved socialization in puppies (14–18). The quality of published research evidence in support of all of these indications is variable, as is the quality of some of the criticisms made concerning the evidence base. For example, the skeptical review of Frank et al. (19) is often cited in the context of an “evidence-based argument” [e.g., (20, 21)] without equal consideration given to the flaws in this latter work which have been highlighted by both Pageat et al. (22) and Beck (23) in the same journal. Both proponents and opponents to the use of pheromones may selectively cite the literature in favor of their case.

There are very few peer reviewed studies on CAP, however initial results are potentially promising regarding its use in reducing inter-cat conflict in multi-cat households. A case report on the use of Feliway Friends^TM^ in a single multi-cat household documents increased cat-cat proximity, increased tolerance, faster recovery following aversive encounters, and increased duration of sleeping in proximity between cohabiting pairs of cats (24). A randomized, double-blind placebo controlled study of 45 households claims a greater decrease in aggression scores with Feliway Friends^TM^ compared with placebo (25). This study is described as a “pilot study” perhaps because an atypical significance threshold was chosen to illustrate a more consistent effect across time, although a conventional significance threshold (*p* < 0.05) would still indicate superiority of the pheromone product over placebo at days 21 and 42. The consistent positive outcome in these circumstances, as well as the specific behavior changes seen in each context, support the hypothesis that CAP also creates some form of bias toward an increased sense of safety in the environment.

It is worth noting that pheromone products are intended for use as an environmental adjunct to aid relaxation. As pheromone products only form part of the environmental input processed by an individal they can be counteracted by more overt threatening cues, and this might explain why, for many behavioral problems, they are generally recommended in combination with a behavioral modification plan. This may include psychopharmacological intervention when addressing an established behavior problem.

Both Adaptil^TM^ and Feliway Friends^TM^ are described as appeasing pheromones due to their origin and compositional similarities, but currently Feliway Friends^TM^ is specifically marketed for conflicts and aggression whereas Adaptil^TM^ has never been evaluated for use in conflict and is not promoted for use in these circumstances (26). The reason behind the different recommendations of these apparently similar products is not clear, but may be based on an assessment of risk and differences in typical cat and dog social behavior, with cats typically being more avoidant and exclusionary than dogs.

Within the cat-dog relationship, the behavior of the cat may be particularly important. A recent study of 748 mixed species homes found that cats were observed to be uncomfortable with dogs or to threaten dogs more frequently than vice versa (9). The study concluded that “comfortability of the cat” was the most important prognostic indicator of the cat-dog relationship (9), however no indication was given as to whether it is the cat's temperament and behavior which underpins its comfortability, or the dog's. Given that many cats and dogs appear to live together in a state of some tension, but not deep hostility, this situation provides an excellent context in which to evaluate the effect and species-specificity of CAP and DAP, without other behavioral intervention. However, given the lack of information about the nature of the determinants of “cat comfortability” it was not possible to predict which product may be better.

To date there has been no scientific study investigating the potential efficacy of pheromone products in improving the cat-dog relationship in multi-species homes. Therefore, the aim of the current study was to undertake a blinded parallel randomized trial to evaluate and compare the effects of Adaptil^TM^ and Feliway Friends^TM^ on the cat-dog relationship in multi-species households where there was some tension in the relationship between the two species. It was hypothesized that both products would improve the cat-dog relationship, but that there should be some differences between the two products given their different compositions. No *a priori* assumptions were made as to specific effects.

## Materials and Methods

Ethical approval for the study was provided by the delegated authority of the University of Lincoln's College of Science Ethics Committee.

Given the limited previous research on the cat-dog relationship, it was necessary first to design an appropriate survey instrument to capture the clinically important details of the relationship, before undertaking the double blinded parallel randomized study.

### Design of Survey Instrument

Focus groups were used to capture the details of the cat-dog relationship that pet owners felt were most important. Separate focus groups were used to discuss cat behaviors and dog behaviors. The focus groups followed a basic template with group discussion encouraged after each question was asked (for template see Supplementary Material Table 4). Groups ran until redundancy on this topic was achieved (two rounds of focus groups for each species). Transcripts were analyzed and behaviors that were consistently considered to be important indicators of the relationship were identified from their frequency within and between groups. The focus groups recruited individuals with a special interest in the veterinary and/or behavior fields. For the veterinary group, inclusion criteria were ownership of both cat(s) and dog(s) and employment at a veterinary practice. For the behavior group, inclusion criteria were ownership of both cat(s) and dog(s) and at least an MSc level qualification in Clinical Animal Behavior. In order to ensure that the themes which emerged from these were understood by non-specialists, a comprehension assessment was undertaken using an interactive PowerPoint survey of the issues raised (27). This presentation was distributed to six volunteer members of the general public recruited through social media. Specifically, these “lay-volunteers” did not work in an animal-related profession or consider themselves to be experts in the field. These volunteers were also given the opportunity to raise any further issues that may have been missed by the expert focus groups. No further issues were raised. Based on the data collected from these activities a 34-item survey was created (See Supplementary Material Table 1). Seventeen items (10 relating to undesirable interactions and seven to desirable interactions) assessed the frequency of specific cat-dog interactions. The ten undesirable interactions were: cat blocking dog's path; dog chasing cat/cat running away (not in play); dog growling at cat; cat hiding from dog or up high; staring; cat swiping at dog; dog barking at cat; cat hissing at dog; dog interrupting fuss of cat (i.e., approaching and causing disruption when the owners fuss the cat); and cat interrupting fuss of dog. The seven desirable interactions were: playing (both pets enjoying play together); sleeping near each other; dog grooming cat; friendly greeting; cat grooming dog; sharing a bed; and both relaxed in the same room. All of these were scored by the owner using a 5-point Likert frequency scale (Several times a day, Daily, 3–6 times this week, 1–2 times this week, Not this week). Additional items assessed cat comfortability, dog comfortability (both scored subjectively by the owner out of ten), changes in owner routine, and only for households with additional pets, the behavior of these other animals in the household, especially in relation to the focal subjects.

### Parallel Randomized Trial (PRT)

Participants for the PRT were recruited using online groups, social media, and poster advertisements in the waiting rooms of veterinary practices. Criteria for inclusion were that participants had to: be 18 years of age or older; own at least one dog and one cat, each of which spend >40% of their time in the home; feel that there was room for improvement in the relationship between one of their dogs and one of their cats; have a 6 week period in which to complete the trial with no holidays or changes in routine planned; and be willing to complete surveys on a weekly basis. Participants also had to confirm that in the 3 months prior to the trial they had not moved to a new house; nor acquired any new pets; nor started treating their pets with any medications which could influence behavior; nor used any Adaptil^TM^ or Feliway Friends^TM^ products.

During an initial telephone interview, qualitative information was collected describing the cat-dog relationships in each household based on a pre-defined list of questions but with room for owner elaboration. On this basis, the participant's animals' relationships were categorized into the following groups: dog interested—cat fearful; cat interested—dog fearful; indifferent; and avoidant. A suitable location for diffuser placement was also identified at this time: a floor-level plug socket in an area frequented by both pets and not obstructed by furniture or close to open windows/doors. Subjects were then allocated a diffuser based on order of recruitment. All diffusers were visually identical and randomly allocated a number for order of use, in batches of 10 (five of each product). The researchers were blind to the treatment groups as this process was undertaken by an individual otherwise independent of the study.

Data concerning the frequency of specific cat-dog interactions and a score of the owner's perception of their pets' overall level of relaxation were collected weekly using the survey instrument (Supplementary Material Table 1). Two weeks of baseline data were captured since it was thought that owners' observation abilities and thus perceptions of the problem might change with the introduction of the recording sheet. Diffusers were posted to each participant in time for them to be plugged in immediately after completion of the second “baseline” week survey.

Immediately after completion of the second week baseline survey, owners were instructed to plug in the diffuser which they had now been allocated, and surveys were completed weekly for a further 4 weeks. All trials were completed between 29th October 2018 to 5th March 2019.

Initial participant groupings (A vs. B) were revealed to the researchers only after all data had been gathered in order to eliminate the risk of bias during the trial. The researchers remained blinded to the specific identity of the groups (i.e., which product each group had received) until all statistical analysis was complete.

The primary outcome of interest was a change in total undesirable interactions over the test period, with the expectation that these would decrease with pheromone diffuser use. The total undesirable interaction score was calculated as the sum of the Likert-scale scores (0–4 for each) of the 10 undesirable interactions. The secondary outcome of interest was change in total desirable interactions, which were expected to increase with diffuser use. The total desirable interaction score was calculated in a similar way. Evaluation of specific behaviors making up each of these scores was only of interest if there was a significant change in these total scores. Additional outcome measures of interest discussed in this paper are “cat relaxation” and “dog relaxation.” Data from the survey relating to the animals' interactions with the owner or wider demeanor are not presented here as they do not relate to the primary question of interest.

Unsolicited qualitative feedback from the owners was also collated in order to gain a deeper insight into any issues arising.

### Statistical Analysis

Analysis was conducted using IBM SPSS version 25 for Windows. A data management and statistical analysis plan for the undesirable and desirable interaction outcomes was developed *a priori* as follows. Where <5% of data throughout the study was missing for a participant, estimated data were manually imputed based on the average of the values for that variable at the time points immediately before and after the missing time point for that subject. Any participant with more than 5% missing data would have been excluded, but this did not occur: missing responses were infrequent and there were only eight missing data points out of a total of the 3,876 recorded (0.2% missing data).

Demographic data were assessed for significant difference between the two groups using chi squared tests of association for categorical data and Mann-Whitney U tests for measures of duration (age of pets and duration of cohabitation).

For the primary outcome measure, total undesirable interaction scores, data were checked for normality and a repeated measures mixed ANOVA was used accordingly with week and treatment group together with their interaction as fixed factors (28, 29). Greenhouse-Geisser corrections were employed to correct for a lack of sphericity. In order to identify the source of differences for significant results *post-hoc* testing was undertaken using pairwise comparisons. For interpretive purposes, effect sizes were calculated (Partial eta squared).

Further testing to identify which specific behaviors were potentially contributing to the overall change was undertaken by comparing Week 2 scores (last baseline period) with Week 6 scores (end of treatment) for individual behaviors. Week 2 was chosen as a point of reference, since it was felt that this would provide a more conservative estimate, given the potential changes in owner perception while learning to use the weekly survey sheets. This comparison was undertaken using non-parametric tests on each of the 10 undesirable behaviors. In accordance with Feise (30) corrections for multiple testing were not made in the individual behavior analysis as this was designed to help elucidate where significant effects might lie and the risk of being overly conservative was considered to outweigh the risks of false-positive results at this stage of the analysis. To determine if there was a difference between the scores of these items for the two baseline weeks (Weeks 1 and 2), within subjects Wilcoxon tests were used, without correction for multiple testing.

A similar statistical procedure was then used for the secondary outcome measure, desirable interaction scores, with a repeated measures mixed ANOVA used for assessment of total desirable interaction scores followed by non-parametric assessment of individual behavior frequencies.

Since there were two outcomes of interest (desirable and undesirable behavior) the significance threshold for evaluation of each of the related ANOVA models was subject to a Bonferroni correction, and revised to *p* < 0.025.

Data relating to the scoring of comfortability were not normally distributed and so were analyzed using non-parametric analyses. In order to reduce the number of analyses undertaken we considered only the data from the second baseline week (Week 2) and the final treatment week of the study (Week 6). Wilcoxon T-tests for within group analysis with a significance threshold of *p* < 0.025 were used for interpretative purposes given the simultaneous evaluation of two outcomes (relaxation of cat and relaxation of dog).

## Results

Thirty-six participants were enrolled onto the trial, of which two participants failed to complete the trial: one due to acquisition of a new pet, one due to loss of an existing pet during the trial period, leaving 34 subjects for analysis. Details of the data generated at each stage of the study are given in the CONSORT Flowchart in Supplementary Material Table 2. Data are reported only for those subjects remaining in the final analysis. Unsolicited, qualitative comments received in emails alongside the survey responses were generally positive and included: “less chasing and more gentle play”; “my dog was his usual pesky self but the cats were much more chilled with each other and with him”; “the whole household has been more content”; “I have seen (the cat and dog) touch noses a few times which is definitely a new thing”; and “I will be going out to purchase one of each of the diffusers until your results are in.” One participant highlighted a consistent increased proximity between her dog and cat which she felt was not adequately captured by the survey: whilst the cat and dog had always been relaxed in the same room of an evening, the cat historically always chose to sit on a cat tower but following diffuser use began to regularly sit on the edge of the sofa nearer to the dog. There were also reports of behavioral changes not directly related to the cat-dog relationship: a dog reported to be much calmer on walks; and another dog that had always been fearful of males taking treats after a couple of hours where previously he had been reported to hide for most of the day if male visitors arrived.

### Participant Demographics

Results are summarized in Tables 1 and 2.

**Table 1:** Participant demographics.

	**Adaptil diffuser**	**Feliway friends**
	**group**	**diffuser group**
Total participants per group	17	17
**Number of pets per household**		
Multi-cat household	11	9
Multi-dog household	7	6
Multiple cats AND multiple dogs	6	3
in household		
**Sex of pets**		
Dogs—Female: Male	5:12	7:10
Cats—Female: Male	7:10	8:9
**Neuter status**		
Both neutered	12	14
Entire dog/neutered cat	5	3
Entire cat	0	0
**Style of cat-dog relationship**		
Dog interested; cat fearful	9	9
Cat interested; dog fearful	2	4
Indifference	2	0
Avoidance	4	4
**Owner demographics**		
Work in animal related profession	16	13
Female: Male	17:0	16:1

**Table 2:** Age and duration cohabiting of pets (years) in each treatment group.

	**Adaptil**		**Feliway friends**	
	**Mean ± SD**	**Median**	**Mean ± SD**	**Median**
Age of dog	4.69 ± 3.94	3	4.62 ± 4.25	3.5
Age of cat	6.35 ± 3.02	7	6.91 ± 4.47	6
Duration of cohabitation	2.44 ± 2.40	1.5	2.65 ± 1.91	2.5

There were no significant differences between the two diffuser groups in the number of pets per household (multi-cat/multi-dog/both, all *p* > 0.2), the sex of pets (dogs, *p* = 0.47, cats, *p* = 0.73) or the neuter status of pets (*p* = 0.42).

There were no significant differences between the groups in age of cat, age of dog or duration cohabiting (all *p* > 0.5).

Data relating to changes in total desirable and undesirable interaction scores across the weeks of the study are summarized in Table 3, with main effects described in the following sections.

**Table 3:** Mean ± standard deviation total desirable and undesirable interaction scores by week.

**Week**	**Adaptil**		**Feliway friends**	
	**Desirable**	**Undesirable**	**Desirable**	**Undesirable**
1	3.65 ± 2.57	8.94 ± 5.93	5.24 ± 4.84	11.41 ± 5.28
2	3.54 ± 2.12	8.76 ± 5.38	5.00 ± 4.21	10.00 ± 5.91
3	4.24 ± 2.82	6.24 ± 5.65	5.47 ± 3.88	7.53 ± 4.89
4	4.53 ± 2.70	6.18 ± 4.71	5.88 ± 3.88	6.59 ± 4.60
5	4.53 ± 2.55	5.76 ± 4.84	6.59 ± 4.73	7.24 ± 3.90
6	4.41 ± 2.32	5.29 ± 5.18	7.06 ± 5.13	5.88 ± 4.65
**Difference**	**0.88 ± 2.14**	**−3.47 ± 3.87**	**2.06 ± 3.73**	**−4.12 ± 4.04**
**Week 6–2**				

*Shaded rows represent baseline period, bold row describes difference between last baseline week (Week 2) and last week of treatment (Week 6)*.

### Undesirable Behaviors

Between Weeks 2 and 6, ≥50% reduction in undesirable behavior scores was seen in 8/17 (47%) participants receiving Adaptil^TM^ and 5/17 (29%) participants receiving Feliway Friends^TM^. A reduction ≥30% in undesirable behaviors was seen in 12/17 (71%) participants in both groups.

Total undesirable interaction scores significantly differed across the weeks F_(3.3, 105.3)_ = 21.086, *p* < 0.001, η^2^_p_ = 0.397; there was no significant difference between the diffusers F_(1, 32_) = 0.61, *p* = 0.440, η^2^_p_ = 0.02, and no significant interaction between week and diffuser group was observed F_(3.3, 105.3)_ = 0.832, *p* = 0.488, η^2^_p_ = 0.025. *Post hoc* pairwise comparisons showed that there was no difference between Weeks 1 and 2 (baseline period), but that both of these weeks differed from each of Weeks 3–6 (treatment period, all *P* ≤ 0.001).

**Figure 1:**
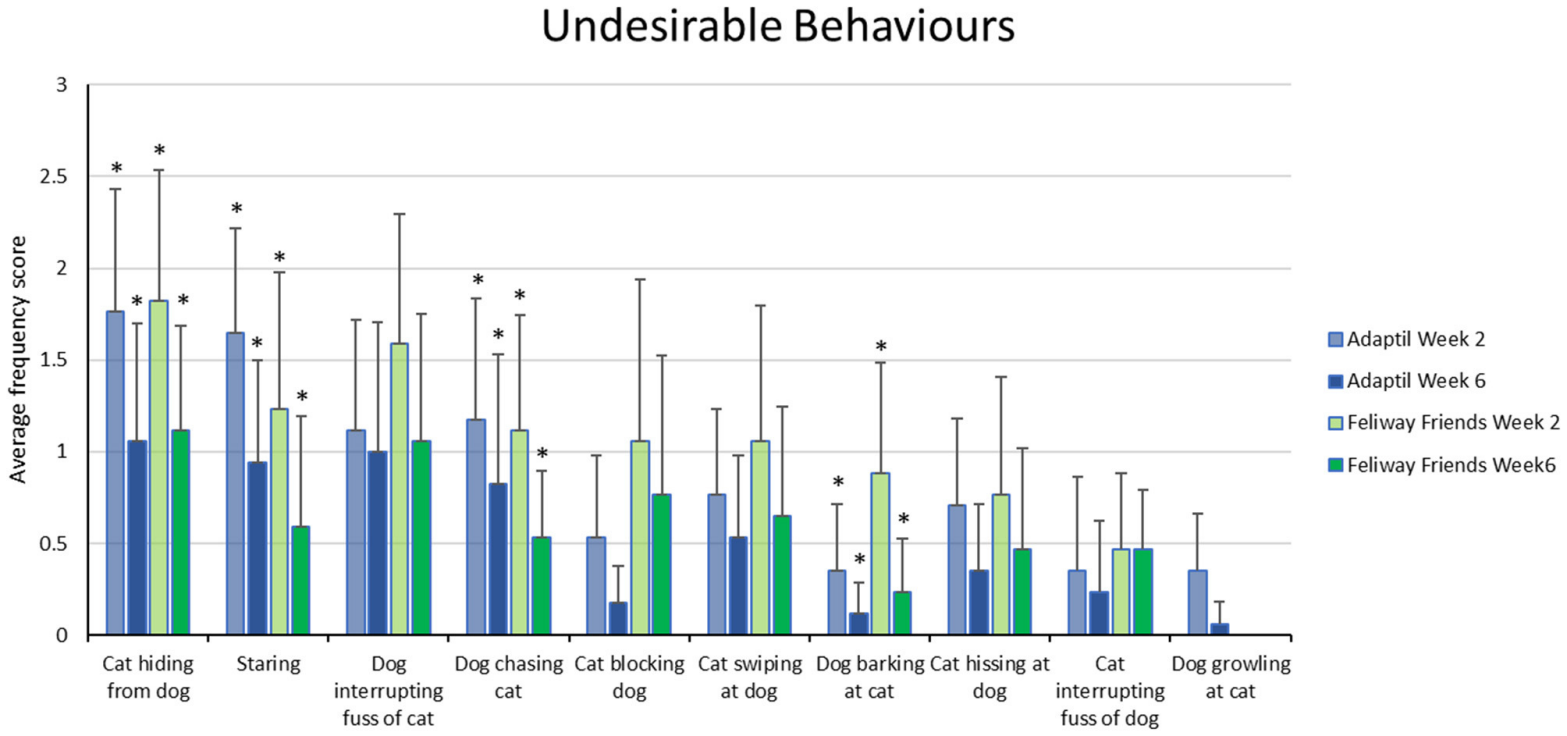
Mean (± 95% confidence interval) behavior frequency scores for individual undesirable interactions at baseline (week 2) and end of trial (week 6). Frequency scores as per Likert scale, 0 = not this week; 1 = once or twice this week; 2 = three to six times this week; 3 = daily; 4 = several times per day. *indicates a significant difference (*p* < 0.05) between related week 2 and week 6 scores.

The data relating to the specific behaviors making up the undesirable interaction score, at baseline (week 2) and at the end of the trial (week 6) are illustrated in Figure 1 below (full dataset available in Supplementary Material Table 3). For those who received Adaptil^TM^, there was a significant decrease in: dog chasing cat/cat runs away (Wilcoxon T = 4.5, *p* = 0.034); cat hiding from dog (T = 3.5, *p* = 0.012); cat/dog staring at the other (T = 3.5, *p* = 0.012); and dog barking at cat (T = 0, *p* = 0.046). For those receiving Feliway Friends^TM^, there was a significant decrease in the same behaviors: dog chasing cat/cat runs away (T = 3.5, *p* = 0.034); cat hiding from dog (T = 0, *p* = 0.026); cat/dog staring at the other (T = 0, *p* = 0.014); and dog barking at cat (T = 0, *p* = 0.014).

Baseline individual undesirable behavior frequency scores did not differ significantly from each other (i.e., Week 1 vs. Week 2 for each of the 10 undesirable behaviors, all *P* > 0.1).

### Desirable Behaviors

Between Weeks 2 and 6, ≥50% increase in desirable interaction scores was seen in 6/17 (35%) participants receiving Adaptil^TM^ and 8/17 (47%) participants receiving Feliway Friends^TM^. A ≥ 30% increase in desirable interaction scores was seen in 8/17 (47%) of participants in both groups.

Total desirable interaction scores differed significantly across the weeks F_(3.531, 112.990)_ = 4.281, *p* < 0.01, η^2^_p_ = 0.118. No significant difference was observed between the two diffuser types F_(1, 32_) = 2.456, *p* = 0.127, η^2^_p_ = 0.071, and no significant interaction between week and diffuser group was observed F_(3.531, 112.990)_ = 0.763, *p* = 0.537, η^2^_p_ = 0.023. *Post hoc* pairwise comparisons did not detect significant differences between any specific pairs of weeks, however analysis of within-subject contrasts showed a significant linear relationship between Week and desirable behavior score F_(1, 32_) = 11.231, *p* < 0.01, η^2^_p_ = 0.260, with a linear increase in total desirable interaction scores from Week 1 to Week 6.

**Figure 2:**
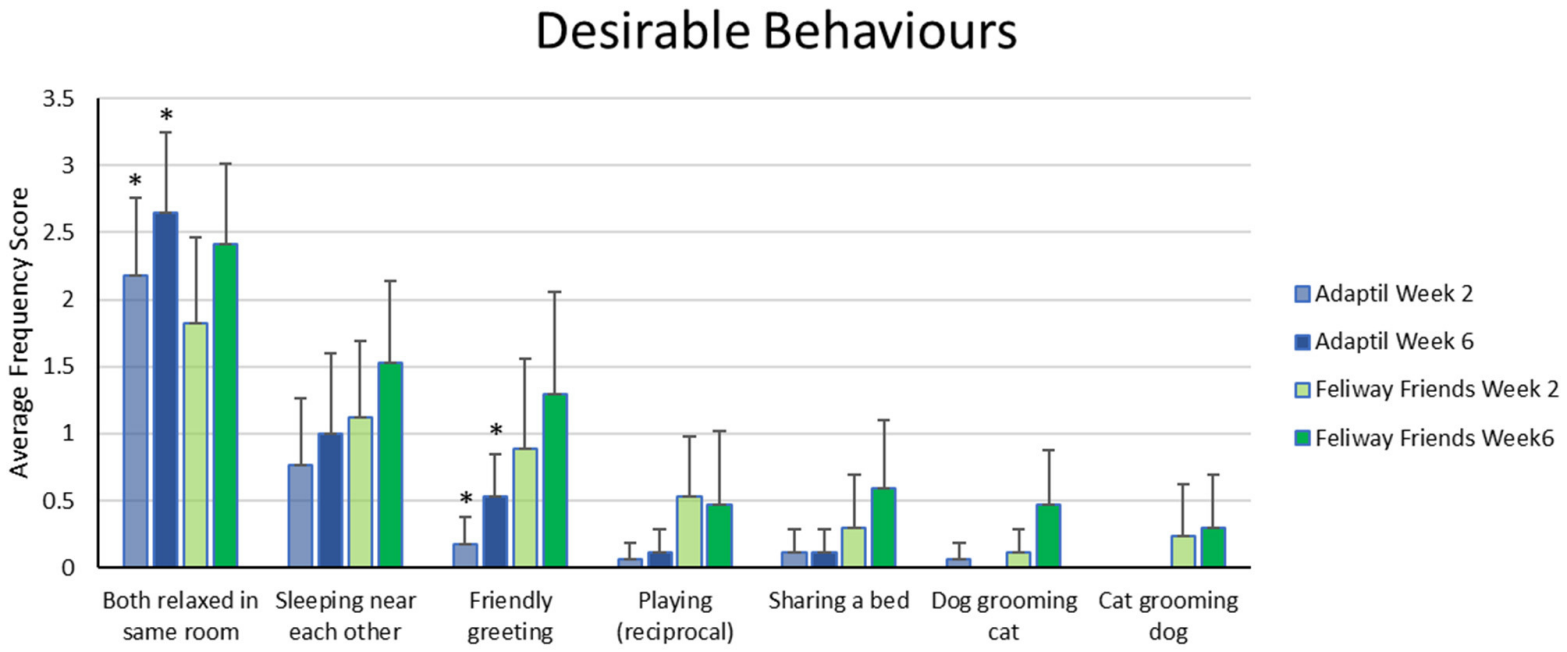
Mean (± 95% confidence interval) behavior frequency scores for individual desirable behaviors at baseline (week 2) and end of trial (week 6). Frequency scores as per Likert scale, 0 = not this week; 1 = once or twice this week; 2 = three to six times this week; 3 = daily; 4 = several times per day. *indicates a significant difference (*p* < 0.05) between related week 2 and week 6 scores.

The data relating to the specific behaviors making up the desirable interaction score, at baseline (week 2) and at the end of the trial (week 6) are illustrated in Figure 2 below (full data available in Supplementary Material Table 3). For those who received Adaptil^TM^, there was a significant increase in: friendly greeting (T = 15, Z = −2.121, *p* = 0.034) and frequency of times spent relaxed in the same room (T = 32.5, Z = −2.126, *p* = 0.033). For those who received Feliway Friends^TM^, none of the individual desirable behaviors demonstrated a significant change from Week 2 to Week 6.

Baseline individual desirable behavior frequency scores did not differ significantly from each other (i.e., Week 1 vs. Week 2 for each of the seven desirable behaviors, all *P* > 0.1).

### Cat and Dog Relaxation Scores (Table 4)

In the Adaptil^TM^ treatment group there was no significant (adjusted *p* < 0.025) difference in cat relaxation scores from baseline (Week 2) to the end of the trial (Week 6) (T = 66.5, *p* = 0.028) but a significant difference was seen in dog relaxation scores (T = 4.0, *p* = 0.021). Conversely, in the Feliway Friends^TM^ treatment group there was no significant difference in dog relaxation scores from baseline (Week 2) to the end of the trial (Week 6) (T = 40.0, *p* = 0.037) but a significant difference was found in cat relaxation scores between Week 2 and Week 6 (T = 71.0, *p* = 0.011).

**Table 4:** Mean ± standard deviation cat and dog relaxation scores by week.

**Week**	**Adaptil**		**Feliway friends**	
	**Cat relaxation**	**Dog relaxation**	**Cat relaxation**	**Dog**
				**relaxation**
1	6.71 ± 1.21	7.94 ± 1.03	6.24 ± 1.95	7.59 ± 2.09
2	6.77 ± 1.56	7.77 ± 1.15	6.77 ± 1.86	7.47 ± 1.81
3	7.82 ± 1.13	8.24 ± 1.09	7.71 ± 1.65	8.18 ± 1.67
4	7.71 ± 1.16	8.29 ± 1.31	7.41 ± 1.94	8.35 ± 1.37
5	7.71 ± 1.45	8.18 ± 1.33	8.12 ± 1.17	8.77 ± 0.90
6	7.88 ± 1.32	8.29 ± 1.16	8.12 ± 1.69	8.59 ± 1.28
**Difference**	**1.11 ± 1.73**	**0.52 ± 0.80**	**1.35 ± 1.87**	**1.12 ± 2.00**
**Week 6–2**				

*Shaded rows represent baseline period, bold refers to last baseline vs. last treatment week difference*.

## Discussion

There was a beneficial response to diffuser use with a significant decrease in undesirable interaction scores and a significant increase in desirable interaction scores across the trial period, regardless of the specific product used. By contrast, improved relaxation scores at the end of treatment compared to baseline, were species-specific. Response to treatment appeared to be rapid with the greatest decrease in undesirable behavior scores occurring during the first week of diffuser use. Partial η^2^ values of above 0.14 are traditionally considered large, so the effect of the two treatments on undesirable behavior is very large, while the effect on desirable behavior is medium.

There are two obvious possible interpretations of the absence of a significant difference between the treatment groups in these outcomes: either both groups were subject to a similar placebo effect; or both diffuser products were similarly effective.

The placebo effect is greater when subjects are aware that there is a higher likelihood of receiving an active treatment in a trial, such as in comparative study designs like the current trial (31). For example, in an analysis of 48 placebo-controlled vs. 42 comparator studies on human treatments for depression, a 15% higher response rate for a particular medication was found in the comparator trials (32). Even so, the high response rate seen in the current trial, is beyond that expected for a placebo even in a comparative trial (32). Steps were also taken in the current trial to reduce owner subjectivity with the desirable and undesirable interactions assessed from relatively objective measures of observation frequency (from never to several times per day) of specific events rather than with some form of subjective rating scale. Further evidence against the results being those of a placebo, comes from the differential effect reported on cat/dog relaxation scores, which were consistent with predicted product-specific effects. Thus, it seems that the common components to these products affect the tendency for undesirable interactions, but the species-specific elements may affect comfortability or relaxation of the individual in the presence of another. This hypothesis deserves further investigation.

The two groups were well matched and thus the different effects noted between the groups cannot be obviously attributed to any demographic factor; so the difference in the products offers the most parsimonious explanation.

Whilst it might be expected that Feliway Friends^TM^ would be more effective in multi-species homes given the apparently stronger contribution of the cat's comfortability to the quality of the cat-dog relationship (9), this did not appear to be the case. Indeed, although there were no statistically significant differences in effect between the products overall on either total undesirable or desirable interaction scores, only the dog-specific product had a significant effect on increasing specific desirable behaviors. We suggest this apparent contradiction might be explained by the behavior of the dog being the primary determinant of the cat's quality of interaction with it. For example, a more relaxed dog may be less likely to disturb the cat (e.g., by chasing it), resulting in a cat that is less stressed and more willing to form some form of social bond with the dog, increasing the desirable interactions perceived alongside a reduction in undesirable interactions. By contrast a more relaxed cat may reduce the likelihood of undesirable interactions, but not necessarily increase desirable interaction so much as the dog may still engage in unacceptable interactions from the cat's perspective, even if they are playful from the dog's perspective and seen as acceptable by the owner. This is in keeping with the demographic split in this study, which showed that over half of the relationships at baseline could be described as “dog interested—cat fearful.” It is likely that the cat's comfortability is indeed important, but that excessive interest from the dog may prohibit the cat from feeling comfortable around him.

Relationship categories of amicable, aggressive or indifferent have been suggested by Feuerstein and Terkel (7), but these do not consider which is the aggressor/victim in “aggressive” relationships, nor allow for relationships in which pets are fearful, but actively avoid one another. For the purpose of this study, descriptions of relationships were taken at induction and relationships were grouped into four categories: dog interested—cat fearful; cat interested—dog fearful; indifferent; and avoidant (there were no purely amicable relationships as these would not have met the inclusion criteria). Although ratios were similar between groups (Adaptil^TM^ 9:2:2:4 and Feliway Friends^TM^ 9:4:0:4) categorization was not straightforward: there was some overlap between categories and variation in the style of the behaviors described within categories. In particular, fearful cats were typically described as running away or showing aggressive behaviors such as hissing/ swiping, but often showed combinations of both, preventing separation into fearful-flee and fearful-aggressive categories. Nonetheless we propose that this classification system provides a useful basis for future studies. There is undoubtedly a need for more basic ethological research to describe cat-dog interactions and how these might group into particular styles of behavior, as has recently been done with separation related problems in dogs (33).

From a practical clinical perspective, client perceptions and feedback are very important, since they determine both the size of the problem but also the feasibility of the treatment. Overall, feedback from the trial was positive with participants reporting that they found the experience enjoyable and the survey straightforward to complete. Accordingly, we suggest that the survey instrument developed here (Supplementary Material Table 1) be used as a routine method of monitoring homes with this problem. The subtle, unsolicited observations of the behavior of pets in the home reported by some clients were an unexpected bonus, which may also have been facilitated by the use of the survey instrument to encourage closer observation of the pets in the home.

Unfortunately, the survey item which asked participants to decide whether the cat-dog relationship was “the same,” “better” or “worse” than normal each week had to be excluded from analysis as it was found to be ambiguous. The ambiguity arose as it was not clear to some participants whether a consistent improvement with no new changes from when the diffuser had first been plugged in should be marked as “the same” (as the previous week) or “better” (compared with baseline). For future research it is advised to replace this item with a ten-point scale rating of the quality of the relationship each week, such that changes in the relationship across previous weeks do not impact the participant's rating.

It would be useful to further study the efficacy of these pheromone products in multi-species households using the revised survey instrument with a larger sample size and a four-group set up, with two diffusers per household comprising: placebo-placebo; placebo-Adaptil^TM^; placebo-Feliway Friends^TM^; Adaptil^TM^-Feliway Friends^TM^. It is possible that the use of both products together could increase efficacy since each product has a different composition and, as demonstrated here, efficacy profile. There are no data that the two products counteract each other, and the manufacturer says both can be used in the home together.

Six participants (18%) subjectively reported no change at all with the diffuser use, and these responses corresponded quite well with the changes in undesirable behavior scores seen, with five of these participants seeing <30% decrease in undesirable behaviors: two had small decreases in undesirable behavior scores, two had no change at all, and one reported a slight increase in undesirable behaviors. Reviewing participant information for these individuals did not reveal any unequivocal predictive factor, although it should be noted that five of the six (83%) were multi-cat households, whereas only 15 out of the other 28 (54%) of the other households had multiple cats. A predominance of multi-cat households in this study obscured the ability to detect changes in a specific cat-dog relationship, as cat-cat interactions are likely to also have impacted on cat-dog interactions and vice versa. This may however be more representative of the more common situation encountered in practice where multi-cat and multi-dog households are common. The cat-dog relationships were classified as “dog interested, cat fearful” in five cases of these poor responders (83%) and while this was the most commonly reported relationship it had a prevalence of just 46% (13/28) among the remaining participants, the other case was classified as “avoidant” and thus had a similar prevalence to the remaining population (1/6 = 17%, 7/28 = 25%). Further research using larger sample sizes to potentially tease out predictive effects is warranted.

Unsolicited qualitative feedback provided a further richness to the data that helps understand the effect. In this regard comments like: “less chasing and more gentle play”; “my dog was his usual pesky self but the cats were much more chilled with each other and with him”; “the whole household has been more content,” are very consistent with the idea that the chemical signal provides increased reassurance within the environment to the subjects, are non-sedating and not simply anxiolytic like a pharmacological agent.

## Conclusion

Feliway Friends^TM^ and Adaptil^TM^ both improved cat-dog relationships in multi-species households. Although careful attention to the exact interactions affecting the relationship may help to determine the appropriate product, this appears to be of marginal clinical relevance in most cases. From a practical perspective, the evidence suggests that, in the absence of detailed behavioral assessment either appeasing pheromone diffuser product may be used with reasonable expectations of success in multi-species households experiencing tension between subjects. Further study is warranted to assess whether both products could be used together to maximize efficacy since each product has a different formulation and there is no evidence of the two products counteracting each other.

## Data Availability Statement

All datasets presented in this study are included in the article/Supplementary Material.

## Ethics Statement

The study was reviewed and approved by the delegated authority for MSc research of the University of Lincoln's College of Science Ethics Committee. The patients/participants provided their written informed consent to participate in this study and their comments to be quoted anonymously.

## Author Contributions

MP and DM contributed conception and design of the study. MP collected all data, performed the statistical analysis, and wrote the first draft of the manuscript. DM wrote sections of the manuscript and developed the statistical analysis plan. All authors contributed to the article and approved the submitted version.

## Conflict of Interest

The authors declare that the research was conducted in the absence of any commercial or financial relationships that could be construed as a potential conflict of interest.
