# Supplementary material for: Cats vs. Dogs: The Efficacy of Feliway FriendsTM and AdaptilTM Products in Multispecies Homes
Source: Front Vet Sci. 2020 Jul 10;7:399. doi: 10.3389/fvets.2020.00399 (PMC7366870; doi:10.3389/fvets.2020.00399)
Supplement: Supplementary file 1 [file Table_1.DOCX]

**Supplementary material 1: survey, developed following focus group input, as used in the parallel randomised trial**

**Questionnaire: Week X Name:**

If you have multiple cats and/ or dogs, this part of the questionnaire should be completed for the individual cat and dog of most concern.

1. **How often (**and where indicated**, for how long) have you seen each of the following occur between the cat and dog in question?**

Please place an “X” in the one box that most closely applies to the situation, for example if your cat greets your dog with a nose touch every morning, you should place an “X” in daily.

For those where it applies, please also estimate the longest duration for which the given activity has occurred this week, for example if your cat has slept near your dog twice this week, on one occasion for 30 minutes and on another occasion for 2 hours, you should put an “X” in “1-2 times this week” and then write “2 hours” in the “longest duration” box.

|  | **Activity** | **Several times a day** | **Daily** | **3-6 times this week** | **1-2 times this week** | **Not this week** | **Longest duration (time)** |
| --- | --- | --- | --- | --- | --- | --- | --- |
| A | Cat blocking the dog’s path |  |  |  |  |  |  |
| B | Cat interrupting when you were fussing the dog |  |  |  |  |  |  |
| C | Playing (both pets enjoying play together) |  |  |  |  |  |  |
| D | Dog chasing cat/ cat running away (not in play) |  |  |  |  |  |  |
| E | Dog growling at cat |  |  |  |  |  |  |
| F | Cat and dog sleeping near each other |  |  |  |  |  |  |
| G | Cat hiding from dog or choosing to stay up high |  |  |  |  |  |  |
| H | Dog grooming cat |  |  |  |  |  |  |
| I | Cat/ dog staring at the other |  |  |  |  |  |  |
| J | Cat swiping at dog |  |  |  |  |  |  |
| K | Friendly greeting such as nose touch between cat and dog |  |  |  |  |  |  |
| L | Cat grooming dog |  |  |  |  |  |  |
| M | Dog barking at cat |  |  |  |  |  |  |
| N | Cat and dog sharing a bed |  |  |  |  |  |  |
| O | Cat hissing at dog |  |  |  |  |  |  |
| P | Dog and cat both relaxed in the same room |  |  |  |  |  |  |
| Q | Dog interrupting when you were fussing the cat |  |  |  |  |  |  |

1. **Thinking only about this cat’s behaviour, how often have you seen the following occur?**

Please place an “X” in the one box that most closely applies, for example if you have seen your cat playing 4 times this week, place an “X” in “3-6 times this week”.

For those where it applies please also indicate the longest duration for which the given activity has occurred this week, for example if your cat has slept on your lap every evening this week, on most occasions for about an hour and the longest time this week was 3 hours whilst you watched a film, you should put an “X” in “daily” and then write “3 hours” in the “longest duration” box.

|  | **Activity** | **Several times a day** | **Daily** | **3-6 times this week** | **1-2 times this week** | **Not this week** | **Longest duration (time)** |
| --- | --- | --- | --- | --- | --- | --- | --- |
| A | Coming to greet you |  |  |  |  |  |  |
| B | Snuggling up with you or sleeping on your lap |  |  |  |  |  |  |
| C | Laying stretched out on back or side |  |  |  |  |  |  |
| D | Playing with toys |  |  |  |  |  |  |

1. **Overall, on a scale of zero (not relaxed at all) to ten (very relaxed), how relaxed do you think this cat has been this week?**

**0** □ **1** □ 2 □ 3 □ 4 □ 5 □ 6 □ 7 □ 8 □ 9 □ 10 □

**Not at all relaxed Moderately relaxed Very relaxed**

**________________________________________________________________________________________**

1. **Thinking only about this dog’s behaviour, how often have you seen the following occur?**

|  | **Activity** | **Several times a day** | **Daily** | **3-6 times this week** | **1-2 times this week** | **Not this week** | **Longest duration (time)** |
| --- | --- | --- | --- | --- | --- | --- | --- |
| A | Coming to greet you |  |  |  |  |  |  |
| B | Snuggling up with you or sleeping on your lap |  |  |  |  |  |  |
| C | Laying stretched out on back or side |  |  |  |  |  |  |
| D | Playing with toys |  |  |  |  |  |  |

1. **Overall, on a scale of zero (not relaxed at all) to ten (very relaxed), how relaxed do you think this dog has been this week?**

**0** □ **1** □ 2 □ 3 □ 4 □ 5 □ 6 □ 7 □ 8 □ 9 □ 10 □

**Not at all relaxed Moderately relaxed Very relaxed**

1. **How many times this week have you felt the need to intervene to break up an interaction between this dog and cat?**

**None this week** □  **Once or twice** □ **Three or more times** □

1. **Overall, do you think that interactions between your dog and cat have been the same as normal, worse than normal, or better than normal this week?**

**Worse** □  **Normal** □ **Better** □

1. **How much time have you spent at home this week to observe your cat and dog?**

**More than usual** □ **Normal routine** □ **Less than usual** □

**_______________________________________________________________________________________**

**ONLY complete the section below if you have multiple cats or multiple dogs: if you have one of each cat and dog you do not need to complete this section**

**___________________________________________________________________________________**

1. **Overall, on a scale of zero (not relaxed at all) to ten (very relaxed), how relaxed do you think all of your cats have been this week?**

**0** □ **1** □ 2 □ 3 □ 4 □ 5 □ 6 □ 7 □ 8 □ 9 □ 10 □

**Not at all relaxed Moderately relaxed Very relaxed**

1. **Overall, on a scale of zero (not relaxed at all) to ten (very relaxed), how relaxed do you think all of your dogs have been this week?**

**0** □ **1** □ 2 □ 3 □ 4 □ 5 □ 6 □ 7 □ 8 □ 9 □ 10 □

**Not at all relaxed Moderately relaxed Very relaxed**

1. **How many times this week have you felt the need to intervene to break up an interaction between any of your dogs and any of your cats?**

**None this week** □  **Once or twice** □ **Three or more times** □

1. **Overall, do you think that interactions between all of your dogs and cats have been the same as normal, worse than normal, or better than normal this week?**

**Worse** □  **Normal** □ **Better** □

**Thank you for taking the time to complete this questionnaire!**
