# Supplementary material for: Cats vs. Dogs: The Efficacy of Feliway FriendsTM and AdaptilTM Products in Multispecies Homes
Source: Front Vet Sci. 2020 Jul 10;7:399. doi: 10.3389/fvets.2020.00399 (PMC7366870; doi:10.3389/fvets.2020.00399)
Supplement: Supplementary file 2 [file Table_2.DOCX]

**Supplementary material 2: CONSORT Flow Diagram**


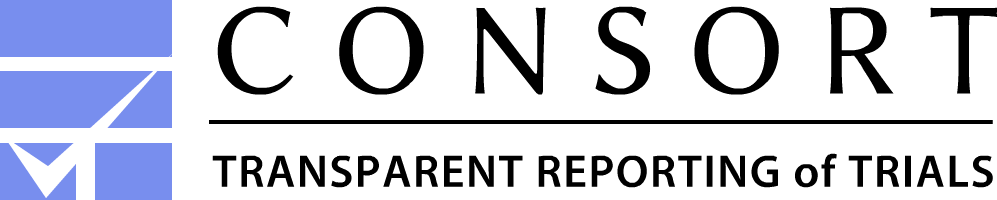


**CONSORT 2010 Flow Diagram**

Lost to follow-up (give reasons) (n= 0)

Discontinued intervention (give reasons) (n= 1)

Reason: acquired another cat mid-study so voluntarily withdrew as no longer met criteria

Analysed (n= 17)
♦ Excluded from analysis (give reasons) (n= 1)

Reason: death of dog in household during Week 6 of study

## Analysis

Analysed (n= 17)
♦ Excluded from analysis (give reasons) (n= )

Lost to follow-up (give reasons) (n= 0)

Discontinued intervention (give reasons) (n= 0)

## Follow-Up

## Enrolment

Allocated to intervention (n= 17)

♦ Received allocated intervention (n= 17)

♦ Did not receive allocated intervention (give reasons) (n= 0)

## Allocation

Allocated to intervention (n= 19)

♦ Received allocated intervention (n= 19)

♦ Did not receive allocated intervention (give reasons) (n= 0)

Randomized (n= 36)

Excluded (n= 5)

♦  Not meeting inclusion criteria (n= 5)

♦  Declined to participate (n= 0)

♦  Other reasons (n= 0)

Assessed for eligibility (n= 41)
