# Supplementary material for: Cats vs. Dogs: The Efficacy of Feliway FriendsTM and AdaptilTM Products in Multispecies Homes
Source: Front Vet Sci. 2020 Jul 10;7:399. doi: 10.3389/fvets.2020.00399 (PMC7366870; doi:10.3389/fvets.2020.00399)
Supplement: Supplementary file 3 [file Table_3.DOCX]

**Supplementary material 3: descriptive statistics, individual behaviours at baseline (week 2) and end of trial (week 6)**

| **Behaviour** | **Adaptil** | | | | | | **Feliway Friends** | | | | | |
| --- | --- | --- | --- | --- | --- | --- | --- | --- | --- | --- | --- | --- |
|  | **Week 2** | | | **Week 6** | | | **Week 2** | | | **Week 6** | | |
|  | **Mean** | **SD** | **Med** | **Mean** | **SD** | **Med** | **Mean** | **SD** | **Med** | **Mean** | **SD** | **Med** |
| Cat blocking dog | 0.53 | 0.874 | 0 | 0.18 | 0.393 | 0 | 1.06 | 1.713 | 0 | 0.76 | 1.480 | 0 |
| Cat interrupting fuss of dog | 0.35 | 0.996 | 0 | 0.24 | 0.752 | 0 | 0.47 | .800 | 0 | 0.47 | .624 | 0 |
| Playing | 0.06 | 0.243 | 0 | 0.12 | 0.332 | 0 | 0.53 | .874 | 0 | 0.47 | 1.068 | 0 |
| Dog chasing cat/ cat runs away | 1.18 | 1.286 | 1 | 0.82 | 1.380 | 0 | 1.12 | 1.219 | 1 | 0.53 | .717 | 0 |
| Dog growling at cat | 0.35 | 0.606 | 0 | 0.06 | 0.243 | 0 | 0.00 | .000 | 0 | 0.00 | .000 | 0 |
| Sleeping near each other | 0.76 | 0.970 | 0 | 1.00 | 1.173 | 1 | 1.12 | 1.111 | 1 | 1.53 | 1.179 | 2 |
| Cat hiding from dog/ up high | 1.76 | 1.300 | 2 | 1.06 | 1.249 | 1 | 1.82 | 1.380 | 2 | 1.12 | 1.111 | 1 |
| Dog grooming cat | 0.06 | 0.243 | 0 | 0.00 | 0.000 | 0 | 1.12 | .332 | 0 | 0.47 | .800 | 0 |
| Cat/ dog staring at the other | 1.65 | 1.115 | 2 | 0.94 | 1.088 | 1 | 1.24 | 1.437 | 1 | 0.59 | 1.176 | 0 |
| Cat swiping at dog | 0.76 | 0.903 | 1 | 0.53 | 0.874 | 0 | 1.06 | 1.435 | 0 | 0.65 | 1.169 | 0 |
| Friendly greeting | 0.18 | 0.393 | 0 | 0.53 | 0.624 | 0 | 0.88 | 1.317 | 0 | 1.29 | 1.490 | 1 |
| Cat grooming dog | 0.00 | 0.000 | 0 | 0.00 | 0.000 | 0 | 0.24 | .752 | 0 | 0.29 | .772 | 0 |
| Dog barking at cat | 0.35 | 0.702 | 0 | 0.12 | 0.332 | 0 | 0.88 | 1.166 | 0 | 0.24 | .562 | 0 |
| Cat and dog sharing a bed | 0.12 | 0.332 | 0 | 0.12 | 0.332 | 0 | 0.29 | .772 | 0 | 0.59 | 1.004 | 0 |
| Cat hissing at dog | 0.71 | 0.920 | 0 | 0.35 | 0.702 | 0 | 0.76 | 1.251 | 0 | 0.47 | 1.068 | 0 |
| Both relaxed in the same room | 2.18 | 1.131 | 2 | 2.65 | 1.169 | 3 | 1.82 | 1.237 | 2 | 2.41 | 1.176 | 3 |
| Dog interrupting fuss of cat | 1.12 | 1.166 | 1 | 1.00 | 1.369 | 0 | 1.59 | 1.372 | 1 | 1.06 | 1.345 | 0 |
