# Supplementary material for: Cats vs. Dogs: The Efficacy of Feliway FriendsTM and AdaptilTM Products in Multispecies Homes
Source: Front Vet Sci. 2020 Jul 10;7:399. doi: 10.3389/fvets.2020.00399 (PMC7366870; doi:10.3389/fvets.2020.00399)
Supplement: Supplementary file 4 [file Data_Sheet_1.docx]

**Supplementary Material 1: Focus Group Template**

**Focus Group Transcript – 1. Cat behaviours/ cat interactions with dogs**

Good evening and welcome. Thanks for taking the time to join us to talk about cat behaviour in cat-dog households. My name is Miriam Kaltz and assisting me is Victoria Kingsman. We're both from the University of Lincoln.

Daniel Mills, who heads up the Animal Behaviour Clinic there, would like us to find out a bit more about how your cats behave in the home, especially around the dog. We want to know what you see that tells you the cat and dog are getting on, what behaviours the cat shows if they’re not comfortable, and how you feel cat-dog interactions might be improved. We are having discussions like this with several groups around Lincolnshire.

You were invited because you have got at least one cat and one dog in your home and are living within our catchment area.

There are no wrong answers but rather differing points of view. Please feel free to share your point of view even if it differs from what others have said. Keep in mind that we're just as interested in negative comments as positive comments, and at times the

negative comments are the most helpful.

You've probably noticed the microphone. We're tape recording the session because we don't want to miss any of your comments. People often say very helpful things in these discussions and we can't write fast enough to get them all down.

We will be on a first name basis tonight, and we won't use any names in our reports. You may be assured of complete confidentiality. The reports will be collated to help us design a questionnaire which will be used for further research. The questionnaire will be used in a study assessing the efficacy of a product that we believe will improve dog-cat relationships.

Well, let's begin. We've placed name cards on the table in front of you to help us remember each other's names. Let's find out some more about each other by going around the table. Tell us your name and where you live.

Questions:

1. Think back over the time you’ve had your pets, what is your fondest memory of them together?
2. Tell me about positive experiences you’ve had with your cat?
3. Tell me about negative experiences you’ve had with your cat?
4. When you decide whether your cat is feeling happy, what do you look for?
5. Let’s list these on the flip chart. Can you decide which two you think are most important? And which two are least important?
6. When you decide whether your cat is feeling stressed, what do you look for?
7. Let’s list these on the flip chart. Can you decide which two you think are most important? And which two are least important?
8. I have got some cards here: each one has a behaviour written on it that you may see your cat perform towards your dog. If you saw your cat doing each of these behaviours, what would you think that meant?
9. For each of these behaviours do you think it is more important to look at how many times your cat does it, or how intense the incident is, or how long it goes on for?
10. Out of everything we have discussed today, what do you think is the most important thing in deciding how your cat feels about the dog?

**Focus Group Transcript – 2. Dog behaviours/ dog interactions with cats**

Good evening and welcome. Thanks for taking the time to join us to talk about cat behaviour in cat-dog households. My name is Miriam Kaltz and assisting me is Victoria Kingsman. We're both from the University of Lincoln.

Daniel Mills, who heads up the Animal Behaviour Clinic there, would like us to find out a bit more about how your dogs behave in the home, especially around the cat. We want to know what you see that tells you the dog and cat are getting on, what behaviours the dog shows if they’re not comfortable, and how you feel dog-cat interactions might be improved. We are having discussions like this with several groups around Lincolnshire.

You were invited because you have got at least one cat and one dog in your home and are living within our catchment area.

There are no wrong answers but rather differing points of view. Please feel free to share your point of view even if it differs from what others have said. Keep in mind that we're just as interested in negative comments as positive comments, and at times the

negative comments are the most helpful.

You've probably noticed the microphone. We're tape recording the session because we don't want to miss any of your comments. People often say very helpful things in these discussions and we can't write fast enough to get them all down.

We will be on a first name basis tonight, and we won't use any names in our reports. You may be assured of complete confidentiality. The reports will be collated to help us design a questionnaire which will be used for further research. The questionnaire will be used in a study assessing the efficacy of a product that we believe will improve dog-cat relationships.

Well, let's begin. We've placed name cards on the table in front of you to help us remember each other's names. Let's find out some more about each other by going around the table. Tell us your name and where you live.

Questions:

1. Think back over the time you’ve had your pets, what is your fondest memory of them together?
2. Tell me about positive experiences you’ve had with your dog?
3. Tell me about negative experiences you’ve had with your dog?
4. When you decide whether your dog is feeling happy, what do you look for?
5. Let’s list these on the flip chart. Can you decide which two you think are most important? And which two are least important?
6. When you decide whether your dog is feeling stressed, what do you look for?
7. Let’s list these on the flip chart. Can you decide which two you think are most important? And which two are least important?
8. I have got some cards here: each one has a behaviour written on it that you may see your dog perform towards your cat. If you saw your dog doing each of these behaviours, what would you think that meant?
9. For each of these behaviours do you think it is more important to look at how many times your dog does it, or how intense the incident is, or how long it goes on for?
10. Out of everything we have discussed today, what do you think is the most important thing in deciding how your cat feels about the dog?

**Flash cards**

Staring

Stalking

Chasing

Fleeing

Crouching

Shaking or trembling

Biting

Hissing/ Spitting/ Growling (cats) / Growling (dogs)

Screaming

Tail twitch or lash (cats) Tail wag (dogs)

Blocking

Hiding

Nose-touching

Being in same room

Sleeping together

Grooming

Sharing bed

Sharing food

Playing

**References**

<https://www.eiu.edu/ihec/Krueger-FocusGroupInterviews.pdf>

Hydén, L. and Bülow, P. (2003). Who's talking: drawing conclusions from focus groups—some methodological considerations. *International Journal of Social Research Methodology*, 6(4), pp.305-321.

Jackson, P. (1998). Focus group interviews as a methodology. *Nurse Researcher*, 6(1), pp.72-84.

Kitzinger, J. (1994). The methodology of Focus Groups: the importance of interaction between research participants. *Sociology of Health and Illness*, 16(1), pp.103-121.

Kitzinger, J. (1995). Qualitative Research: Introducing focus groups. *BMJ*, 311(7000), pp.299-302.
