# Supplementary material for: Cats vs. Dogs: The Efficacy of Feliway FriendsTM and AdaptilTM Products in Multispecies Homes
Source: Front Vet Sci. 2020 Jul 10;7:399. doi: 10.3389/fvets.2020.00399 (PMC7366870; doi:10.3389/fvets.2020.00399)
Supplement: Supplementary file 5 [file Data_Sheet_2.docx]

**Supplementary figure 1: Graph of percentage change in undesirable behaviours across the study period**

*Percentage change has been calculated from weekly group mean scores for undesirable behaviours; error bars represent the 95% confidence interval calculated from the individual undesirable behaviour scores.*

**Supplementary figure 2: Graph of percentage change in desirable behaviours across the study period**

*Percentage change has been calculated from weekly group mean scores for desirable behaviours; error bars represent the 95% confidence interval calculated from the individual desirable behaviour scores.*

**Supplementary figure 3: Graph of percentage change in dog relaxation scores across the study period**

*Percentage change has been calculated from weekly group mean scores for dog relaxation; error bars represent the 95% confidence interval calculated from the individual dog relaxation scores.*

**Supplementary figure 4: Graph of percentage change in cat relaxation scores across the study period**

*Percentage change has been calculated from weekly group mean scores for cat relaxation; error bars represent the 95% confidence interval calculated from the individual cat relaxation scores.*
